# Three-Dimensional Rotational Angiography during Catheterization of Congenital Heart Disease – A ten Years’ experience at a single center

**DOI:** 10.1038/s41598-020-63903-x

**Published:** 2020-04-24

**Authors:** Stefanie Söder, Wolfgang Wällisch, Sven Dittrich, Robert Cesnjevar, Jean-Pierre Pfammatter, Martin Glöckler

**Affiliations:** 1 Department of Pediatric Cardiology, University Hospital Erlangen, Erlangen, Germany; 2 Department of Congenital Heart Surgery, University Hospital Erlangen, Erlangen, Germany; 3 Department of Cardiology, Pediatric Cardiology, Inselspital, Bern University Hospital, University of Bern, Bern, Switzerland

**Keywords:** Interventional cardiology, Three-dimensional imaging

## Abstract

This paper aims to assess the usability and advantages of three-dimensional rotational angiography (3DRA) in patients with congenital heart disease (CHD) and its application in the cath lab. Up to now, its use in CHD is not widespread or standardized. We analyzed all patients with CHD who underwent a 3DRA at our facility between January 2010 and May 2019. The 3DRAs were evaluated for radiation exposure, contrast dye consumption, diagnostic utility and image quality. We performed 872 3DRAs. 3DRA was used in 67.1% of the cases for interventional procedures and in 32.9% for diagnostic purposes. Two different acquisition programs were applied. The median dose-area product (DAP) for all 872 rotations was 54.1 µGym^2^ (21.7–147.5 µGym^2^) and 1.6 ml/kg (0.9–2.07 ml/kg) of contrast dye was used. Diagnostic utility of the generated 3D-model was rated superior to the native 3D angiography in 94% (819/872). 3DRA is an excellent and save diagnostic and interventional tool. However, 3DRA has not become a standard imaging procedure in pediatric cardiology up to now. Effort and advantage seems to be unbalanced, but new less invasive techniques may upgrade this method in future.

## Introduction

Congenital heart disease (CHD) is often associated with complex anatomical anomalies. High-resolution imaging modalities are particularly helpful in this context^1^. During the last decade, the three-dimensional rotational angiography (3DRA) has emerged as a new facility for diagnostic and interventional procedures. The 3DRA is performed by a C-arm of the angiography-system equipped with a flat detector. It generates a 3D volume data set from a single C-arm rotation (at least 180° plus fan angle) around the patient during a continuous injection of contrast dye. It provides a precise view of cardiovascular and surrounding structures in various projections^2^ and can be used for 3D-guidance additionally^3,4^.

The 3DRA, also called flat detector computed tomography (FD-CT) or cone beam CT, was developed in the 1990s and initially used for neuroradiology procedures. In 1997, Fahrig *et al*. published the first experience with this technique in the setting of neuroendovascular therapy procedures^5^. Furthermore, the benefit of this imaging tool was described for coil embolization of cerebral aneurysm, cardiac electrophysiology, valve replacement and liver tumor embolization^6–9^.

In 2010, Glatz *et al*. presented the first systematic analysis of 3DRA in the cardiac catheterization laboratory for patients with CHD^10^. The 3DRA allows a real-time viewing of the native angiography (native 3DRA) as well as post processed precise 3D model (reconstructed 3DRA) for diagnostic purpose. Additionally, 3D navigation in catheter-based interventions is feasible. Nowadays, CT-like soft tissue image quality is acquirable due to continuous development of this technique^2^.

Until now, 3DRA has not become a standard procedure in the pediatric cardiac catheterization laboratory. This technique requires X-ray, a high volume contrast injection and is acquired intraprocedural with obstruction of the continuous course of catheterization. Due to the limited temporal resolution, procedures like rapid pacing, breath-holdings or bolus injection of adenosine are in use to optimize image quality with possible side effects^2,3,10–20^. Therefore, we evaluated usability and possible benefit of 3DRA in patients with CHD, particularly with regard to other less invasive technologies that may replace this technique in the future.

## Material and Methods

### Study design and patients

This study was conducted in accordance with the Declaration of Helsinki and the ethics commission of the Friedrich-Alexander-University Erlangen-Nuremberg (Re.-No. 4477) approved the experimental protocol. All patients were complying with our institutional requirements for cardiac catheterization. Parental written informed consent for cardiac catheterization and contrast-enhanced imaging was obtained for all children.

All patients with CHD who underwent a 3DRA in our pediatric cardiac cath lab between January 2010 and May 2019 were retrospectively included. All interventional and diagnostic 3DRAs were analyzed for radiation exposure, contrast consumption and particularly, the diagnostic advantage of the 3D-models achieved by post processing over the native 3D angiography.

### Rotational angiography

All examinations were performed on a biplane angiography system with two 20 × 20 cm^2^ flat-panel detectors (Axiom Artis, Siemens Medical Solutions, Forchheim, Germany). Image acquisition was executed with two different settings. The so-called diagnostic program (5sDRc) was used with 30 frames per second (f/s), a dose of 0.36 µGy per image, 5 s scanning time, with a fixed tube voltage of 90 kV and automatically adapted tube current. In contrast, with the low-dose program (5sDR-L) the tube voltage was reduced to 70 kV, a 0.2 mm copper filter was applied and the dose per image was only 0.1 µGy. The frames per second and scanning time were identical in both programs. The anti-scatter grid was removed in patients with a weight lower than 20 kg.

The native 3DRA is a 5 s angiography recorded continuously while moving the c-arm 180 degrees around the patient. This data is post-processed automatically by a workstation to create a 3D model from the prior angiography (Leonardo DynaCT, InSpace 3D software, Siemens Medical Solutions). It is commercially available. In a further step, the 3D-model was analyzed and modified by a pediatric cardiologist immediately after acquisition. This implies adapting the lightning and opacity settings and cropping of obstructing structures like bones. Cross-sectional images were created with 0.4 mm thickness in a 500 × 500 matrix format. In case of interventions, a 3D model in volume rendering format (VRT) was always merged with the real-time fluoroscopic image of the “A” camera (2D-3D-Registation)^2^. The radiation dose was measured as dose-area product (DAP) in the collimator housing, independent from distance of the patient. The contrast agent Imeron® 350 (350 mg iodine/ml; Bracco Imaging Deutschland GmbH) was diluted in the ratio 1:1 with saline. The places of injection and acquisition time were dependent on the volume of interest (VOI) and hemodynamics^4^. The diagnostic value of the reconstructed 3D model (reconstructed 3DRA) in comparison to the prior native 3DRA, which is the base of the 3D-model, was rated directly post catheterization by the operator. A 3-point scale was used: “Superior”, if the 3D-model showed additional information to native 3DRA, “equal” if it added no supplementary information and “minor”, if it was misleading and not helpful due to artefacts.

### Statistical analysis

Descriptive statistics contain continuous variables. They are expressed as a median Q2 with an interquartile range (Q1-Q3), in which Q1 was the 25th percentile and Q3 was the 75th percentile, or mean ± standard deviation (SD). Categorical variables are presented as numbers and percentages. Statistical analysis was performed using StatPlus (Version v6, AnalystSoft Inc.).

### Research involving human and animal participants

All procedures performed in this study involving human participants were in accordance with the ethical standards of the institutional and/or national research committee and with the 1964 Helsinki declaration and its later amendments or comparable ethical standards. This article does not contain any studies with animals performed by any of the authors.

### Informed consent

Informed consent was obtained from all individual participants included in the study for the primary imaging. This study was positively evaluated by the institutional ethic commission (Re.-No. 4477).

## Results

**Figure 1:**
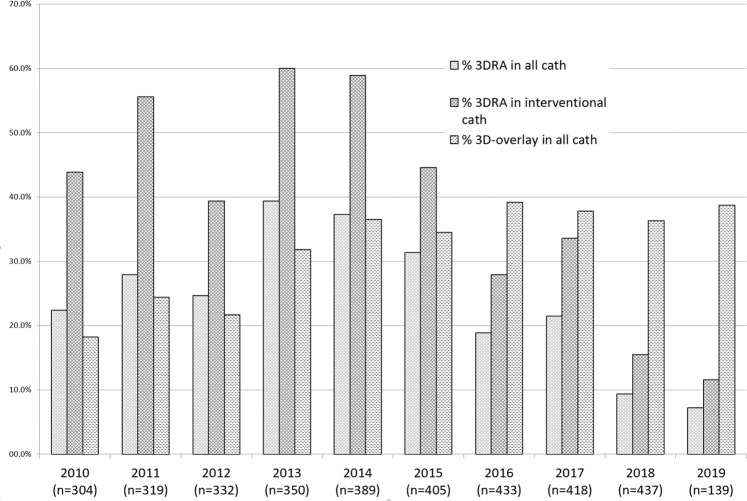
The figure shows the number of all catheterizations and percentage of 3DRAs overall and in interventional procedures in our institution between January 2010 and May 2019. Additionally, the number of all 3D-overlay-procedures, either 3DRA-based or by 2D-3D-registration with models from MRI or CT. The drop of 3DRA in 2014 was caused by an introduction of a new 3D-guidance technique that is based on MRI and CT-datasets.

During the ten years study period, a total of 3526 cardiac catheterizations were performed at our institution. We investigated all patients (n = 507) who underwent a diagnostic or interventional 3DRA in this group. In total, 872 3DRAs were performed in 829 examinations. The percentage of 3DRAs in comparison to all catheterizations is shown in Fig. 1.

67.2% (585/872) of 3DRAs were conducted during interventional procedures and 32.9% (287/872) during diagnostic procedures (Fig. 1). The most common CHD were pulmonary atresia with ventricular septal defect in 12.8% (110/821), aortic coarctation in 12.5% (112/872), double outlet right ventricle in 7.9% (69/872), univentricular hearts after Glenn procedure in 12.4% (108/872) and with Fontan circulation in 5.5% (48/872).

The overall number of 3DRAs peaked in 2014 (n = 145). At the same time, the proportion of low dose 3DRAs (5sDR-L) increased with being our standard acquisition program since 2016. The percentage of 3DRAs in relation to total number of catheterizations was highest in 2013 with 39.4% and decreased continuously to 7.2% in 2019.

The patients had a median age of 3.2 years (0.64–12.46 years) and a median weight of 14.2 kg (7.4–42.5 kg). The demographics of patients and procedural data are shown in Table 1. In 10% of the cases (87/872), the patients were mechanically ventilated during the investigation. No adverse events directly related to the angiography were observed.

**Table 1:** Demographic and procedural data, median (interquartile range), number (percentage of total).

Parameter	Result
Number of patients	507
Male (%)	318 (62.7)
Age (years)	3.5 (0.64–12.46)
Height (cm)	96.0 (69.0–15.0)
Weight (kg)	14.2 (7.4–42.5)
Body surface area (m^2^)	0.6 (0.37–1.34)
Number of catheterizations	829
Number of 3DRAs	872
Diagnostic catheterizations (%)	287 (32.9)
Interventional catheterizations (%)	585 (67.1)
5sDRc (%)	412 (47.2)
5sDR-L (%)	460 (52.8)
3DRA dose-area product (µGym^2^)	54.1 (21.7–147.47)
Total dose-area product (µGym^2^)	240.3 (97.57–692.08)
3DRA contrast-medium/wt (ml/kg)	1.6 (0.9–2.07)
Total contrast-medium/wt (ml/kg)	3.5 (2.15–6.1)

The median amount of contrast agent used for a 3DRA in both protocols was 1.6 ml/kg (0.9–2.07 ml/kg); the median amount of contrast for the entire catheterization was 3.5 ml/kg (2.15–6.1 ml/kg), see Table 2. A continuous administration of contrast agent proximal to VOI facilitates high-resolution images. In most cases, we used a delay time of 1 s to visualize the aorta or 2 s for the pulmonary veins by injection into the pulmonary artery (PA).

**Table 2:** Used contrast medium in our study cohort for 3DRA in ml/kg, median (interquartile range), mean ± SD.

	Diagnostic protocol - 5sDRc (n = 412)	Low-dose protocol - 5sDR-L (n = 460)
Median	1.8 (1.18–2.16)	1.4 (0.77–1.93)
Mean	1.76 ± 0.84	1.45 ± 0.76
Minimum	0.32	0.22
Maximum	5.77	4.03

Overall, median DAP to acquire a 3DRA was 54.1 µGym^2^ (21.7–147.47 µGym^2^). Between 2010 and 2015, the diagnostic program (5sDRc) was used in 412 cases with a median DAP of 73.1 µGym^2^ (48.41–214.63 µGym^2^). Between 2011 and 2018, we utilized the low-dose program (5sDR-L) in 460 cases and the median DAP was even lower with 24.51 µGym^2^ (13.23–97.15 µGym^2^), see Table 3.

**Table 3:** Dose area product in µGym^2^ of 3DRA in our study cohort, median (interquartile range), mean ± SD.

	Diagnostic protocol - 5sDRc (n = 412)	Low-dose protocol - 5sDR-L (n = 460)
Median	73.1 (48.41–214.63)	24.51 (13.23–97.15)
Mean	193.67 ± 263.66	81.17 ± 115.31
Minimum	11.45	1.6
Maximum	1578.3	735.57

On the 3-point scale, we rated the diagnostic value of the reconstructed 3DRA in 94% (819/872) as superior, 5.5% (48/872) as equal and 0.6% (5/872) as inferior to the native 3DRA.

## Discussion

Our systematic analysis of 872 3DRAs shows a conclusive diagnostic value of 3DRA with further substantial advantage of 3D-navigation. To our knowledge, this retrospective single center study is the largest cohort of pediatric cardiology patients in whom 3DRA was applied.

The continuous improvement of 3DRA facilitated the implementation in the pediatric catheterization laboratory^2,10^. This applies especially to certain extracardiac pathologies such as Fontan circulation, aortic arch and pulmonary artery anomalies^2,4,21^. Due to the introduction of a new 3D-guidance modality^22^, the number of 3DRAs decreased continuously since 2014 at our institution. The formation of native 3D angiographies and subsequent 3D-reconstruction seem to be replaceable by other pre-interventional 3D-imaging technologies. This reflects the importance of 3D-imaging and accurate 3D-guidance independent of the matrix used for creation of the 3D models. Figure 1 illustrates the gain of 3D-overlay in our cath lab despite fewer applications of 3DRA.

### Diagnostic utility and image quality

The three-dimensional model derived from 3DRA demonstrates the vascular configuration and the spatial relationship between cardiovascular structures and surrounding tissue very precisely. This becomes particularly clear as we rated the diagnostic value in 94% (819/872) superior to the native 3D angiography. This was due to the reconstructed 3D-models enabling the visualization of anatomy and complex spatial relationships from any desired angle of view. Additionally, comparative measurements of vessel diameters in 3DRAs and biplane angiographies show a high accuracy in settings with a low blood flow (e.g. Cavopulmonary connections) as well as in regions with a more pulsatile flow (e.g. aorta)^3,15,23^. Image quality depends on various factors that can mutually influence each other. The poor temporal acquisition requires a constant injection of contrast dye in the region of interest over seconds with a suitable delay. Moreover, heart movement, spontaneous breathing and the long acquisition time of at least 5 s may result in artefacts with an inferior image quality^2,10,12,18^. To reduce these artefacts, breathholding maneuvers in mechanical ventilated patients are common^2,3,10–12,14–20^. Rapid pacing or injection of adenosine is in use to manipulate heart rate^2,3,13,16,18–20^. Another possibility to achieve a higher image quality is to avoid rapid drainage of the contrast medium by balloon occlusion of the run-off vessel^2^. All these procedures are invasive, with possible side effects. In our institution, we only use breathholding in mechanically ventilated patients.

### Radiation

Our study showed a median DAP of 54.1 µGym^2^ (21.7–147.5 µGym^2^) for all 3DRAs (n = 872). For the last five years, we mainly used the low-dose program (5sDR-L) (n = 460). This resulted in an even lower DAP with a median of 24.5 µGym^2^ (13.2–97.2 µGym^2^). Compared to our previous studies^2,4^, we were able to maintain high quality standard of the images with a significant reduction of radiation dose at the same time. Our results compare well with the study from Aldoss *et al*., which reported a median DAP of 72.3 µGym^2^^12^. Truong *et al*. published similar low radiation doses (DAP = 85 µGym^2^)^17^. Conversely, the study of Haddad *et al*. showed a higher median DAP with 278.0 µGym^2^. This may have resulted from a higher median age (10.2 years) and median weight of the patients (39.8 kg)^20^. A recent study from Goreczny *et al*. presented very high values for both conventional biplane guidance and 3DRA, with significant higher DAP for conventional biplane guidance compared with 3DRA (17745.9 µGym^2^ vs. 10832.3 µGym^2^). This high DAP for 3DRA of 10832.3 µGym^2^ may as well be due to the high median weight of patients (49.3 kg). The small number of patients (n = 6) limits the validity^11^. Berman *et al*. reported about a DAP of 306.0 µGycm^2^ when using 3DRA, which was almost twice that of a biplane angiogram with a DAP of 159.0 µGycm^2^ ^3^. Manica *et al*. have also shown a higher radiation dose with a DAP of 713 µGym^2^ (n = 9) for a 3DRA versus a conventional biplane angiography with 81 µGym^2^ ^24^. However, this large difference was only significant in patients with less than 45 kg bodyweight.

An important factor to assess possible radiation sequels is the effective dose (ED), which considers the individual radiosensitivity of organs^25^. Truong *et al*. described a median ED of 0.16 mSv for 3DRA and compared it to the results of a study population undergoing a pediatric chest CT with an ED of 2.0 mSv for a low-dose protocol^26^. Peters *et al*. focused on ED during 3DRA in children. The median ED was 1.6 mSv (0.7–4.9 mSv) and was calculated with the Monte Carlo method. This work refers to findings of Wielandt *et al*. This group estimated a mean ED of 6.6 ± 1.8 mSV in computer simulated phantoms (PCXMC)^9^. In a phantom study at our facility, ED was calculated in three different anthropomorphic phantoms with a diagnostic and a low-dosage program resulting in an ED for 3DRA lower than 1 mSv^27^.

### Contrast dye

The median amount for a single 3DRA was 1.6 ml/kg (0.9–2.1 ml/kg) in our study. The concentration and amount of contrast dye depends on the anatomic region and hemodynamics. These findings are comparable to those in the reviewed literature. Besides the risk of adverse reactions to iodine carries, the biological effect of radiation is rising with a higher amount of contrast^28^. The great advantage of 3DRA is the fact that one single injection is sufficient to detect any lesion that may be undiagnosed with a conventional biplane angiography^3^. Using 3D-guidance, repeated contrast injections for positioning control can be avoided, which may results in an overall reduction of contrast dye^4,23^. Recently, a study from Goreczny *et al*. compared the 3D navigation modalities: traditional 2D guidance, 3DRA and new software (VesselNavigator) for 2D-3D image fusion in patients who underwent a percutaneous pulmonary valve implantation. They demonstrated a significantly lower contrast use for the VesselNavigator guidance (n = 7) in comparison to 3DRA guidance (1.5 ml/kg vs. 4.7 ml/kg; p = 0.04), but there was no significant difference between the 3DRA guidance and the 2D guidance (p = 0.57)^11^.

### Utility of 3DRA and alternatives

**Figure 2:**
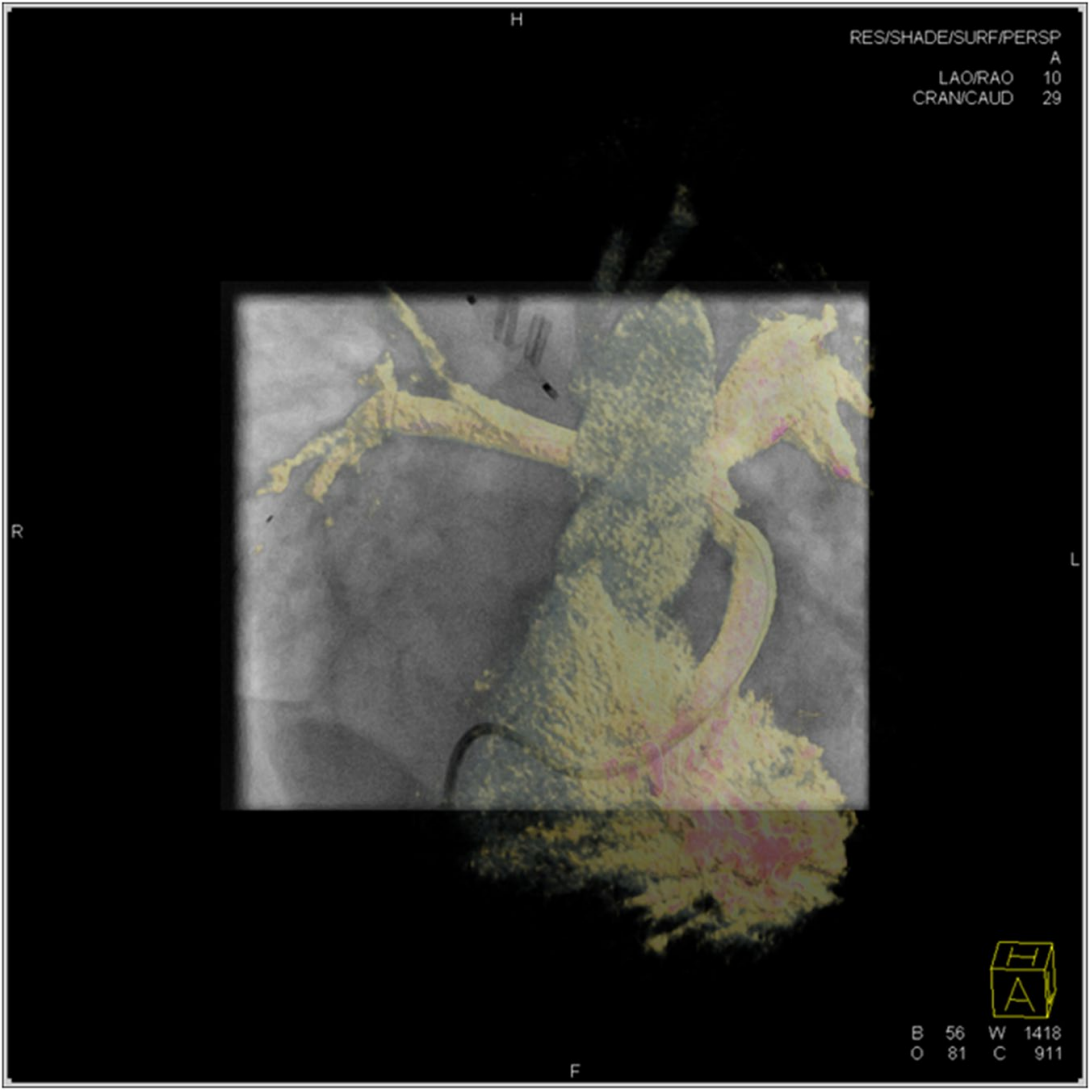
Rotational angiography in the right ventricle in a 3-month-old boy with Hypoplastic left heart syndrome. Overlay in volume rendering format on the fluoroscopic image for 3D guidance. The 3D model may obstruct tiny structures like wires and devices.

The clinical benefit of 3DRA is the generation of high-resolution images, which facilitate understanding and visualization of complex CHD^10^. Furthermore, 3D guidance is a helpful tool in catheter-based interventions as it overcomes specific limitations of conventional biplane angiography (Fig. 2)^4,10,11^.

Patients with complex CHD often undergo various pre-interventional or pre-surgical imaging modalities. Echocardiography is the gold standard in most straightforward cases. Nevertheless, specific issues may be not answered due to a bad acoustic window or difficult tortuous vascular structures^29^. Imaging methods such as CT or MRI have experienced a rapid technical and scientific development in the last decade. Simultaneous to the newly emerging 3DRA, new modalities for CT as the multidetector computed tomography (MDCT) or dual-source computed tomography (DSCT) were established. MDCT has a high spatial and temporal resolution and demonstrates a diagnostic utility that may replace invasive diagnostic methods^30^. The DSCT convinces with a fast subsecond scanning time for the whole thorax acquisition and less radiation exposure (ED less than 0.5 mSv)^31^. A quick generation of 3D data sets by DSCT provides precise anatomical information, making surgical planning easier and more successful^31,32^.

**Figure 3:**
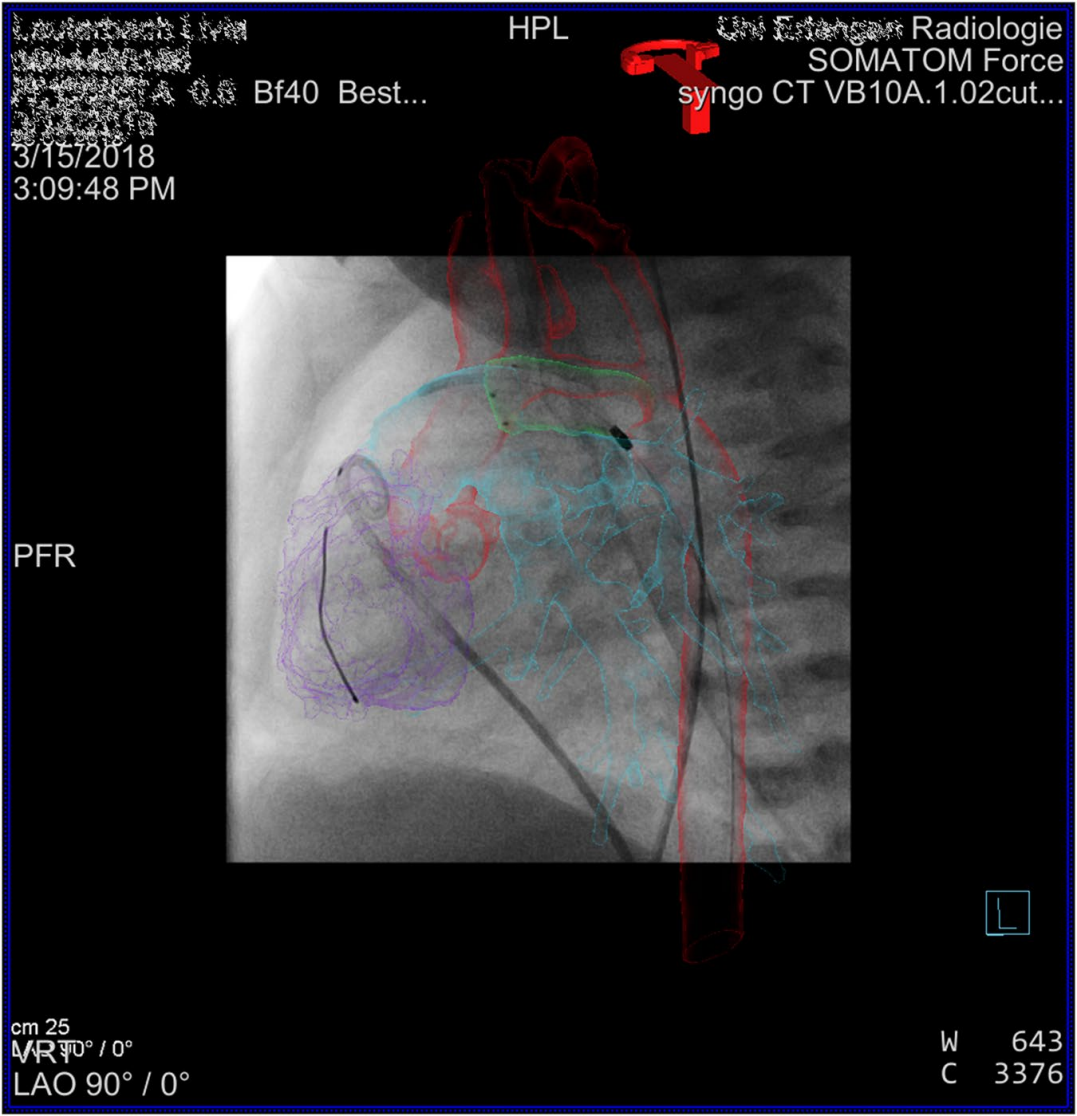
3D-guidance for stenting the arterial duct in a newborn with Hypoplastic left heart syndrome. Basic dataset was a 3^rd^ generation dual source –CT, reconstructed in stereolithographic format. Overlay on both cameras of the biplan angiography machine, shown is the image of the b-camera. The fine delineated model does not obstruct the view on catheters and devices. Color-code light green: ductus arteriosus; red: aorta; violet: right ventricle; light blue: pulmonary artery.

**Figure 4:**
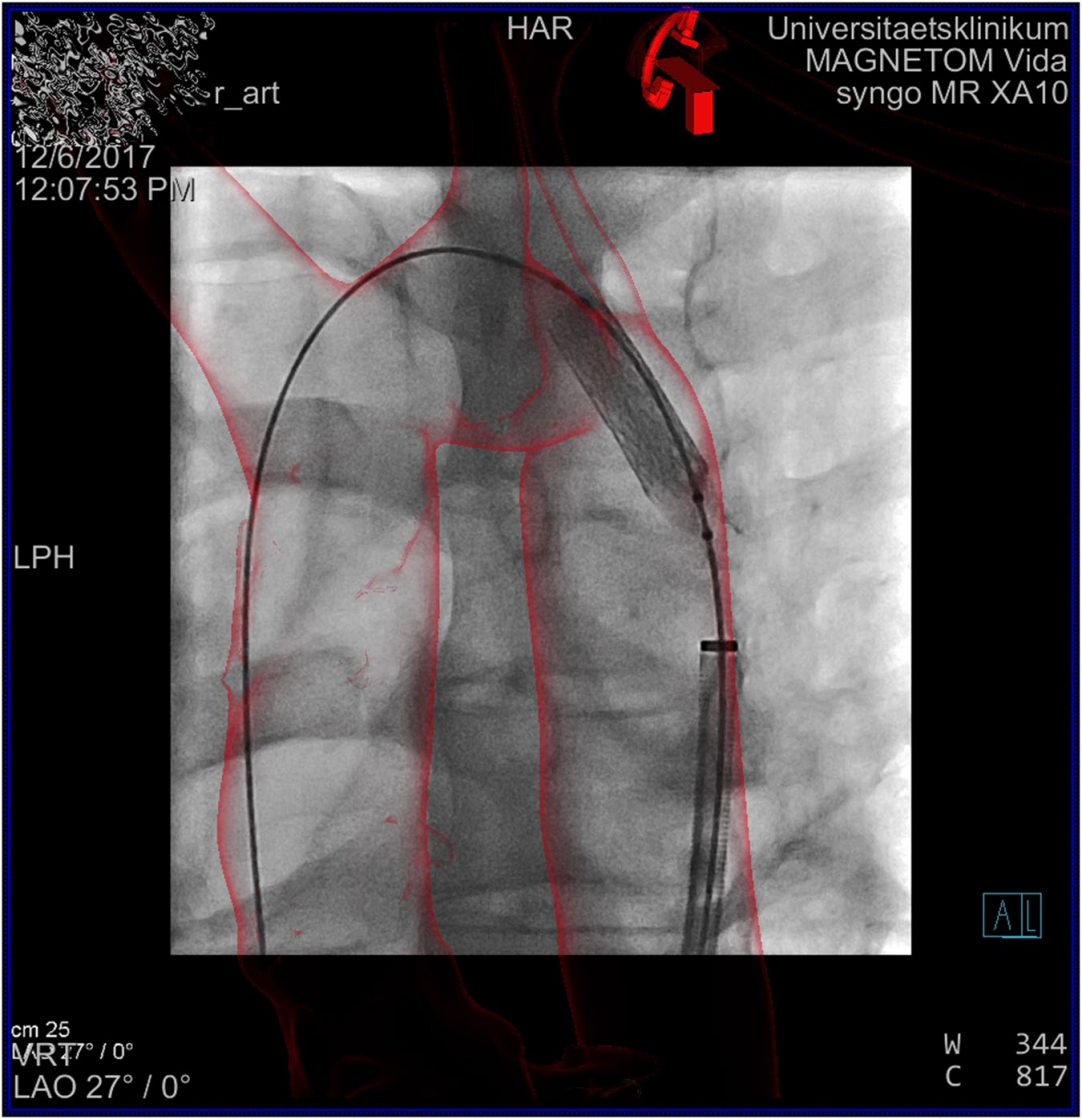
Adult patient with coarctation of the aorta; overlay of a 3D angiography produced by MRI. It is reconstructed in stereolithographic format. 3D-guidance is used for stenting the coarctation.

Recent innovations of 3D-guidance allow 2D-3D fusion of imaging data in different formats without needing images obtained by intraprocedural 3DRA (Figs. 3 and 4). This means further reduction of contrast medium and radiation dose. With these new and innovative imaging tools at hand, some authors concluded that diagnostic 3DRA is negligible and outdated for primary diagnosis and 3D-guidance^22,31^. Additionally, pre-procedural data sets enable an efficient planning of interventional procedures in a timely manner. This is our experience as well and these new developments are leading to a continuous decline of 3DRA in our cath lab since introduction of the new guiding- technologies (Fig. 1).

3DRA is an invasive imaging tool that requires adequate sedation and an vascular access which involves certain risks such as infection, vessel damage and hemorrhage^33^. Poorer resolution in comparison to DSCT is mainly caused by the long acquisition time. Moreover, the generated 3D model from 3DRA has to be processed and visualized in a proper way, which is mostly done immediately after acquisition during cardiac catheterization. This is time consuming, interrupts the course of catheterization and needs special trained staff to adapt the imaging data. From our perspective, 3DRA is still very helpful in unplanned situations during cardiac catheterizations when complex anatomy has to be visualized immediately and accurately as well as in emergencies^2^. A recent study from our faculty demonstrated a new facility of 3D guidance with a 2D-3D registration from previous MRI or CT data sets. We used segmented imaging data in a stereolithographic format (Figs. 3 and 4). That way, we were able to generate superior image quality with much better visibility of catheters and devices compared to traditional image-overlay in a volume rendering format^22^. The possibility to integrate 3D-images from less invasive imaging modalities with pre-operative acquisition reduced the number of 3DRA in our institution significantly. At the beginning, 3DRA being a unique imaging modality for interventional 3D-guidance, we used it in nearly 40% of all catheterizations. Nowadays, it is only used in unplanned catheterization with no existing pre-interventional imaging data or in catheterization with invasive testing. One example is the simultaneous ballooning of the pulmonary conduit and 3DRA of the aortic root to rule out potential coronary compression before transcatheter pulmonary valve replacement.

## Conclusion

3D-imaging improves diagnostic possibilities and quality compared to a native 3DRA which consists out of 2D-projection images. Reconstructed 3DRA are very helpful and well utilizable for 3D-guiding in complex interventions in congenital heart disease. Until now, 3DRA has not become a standard process for diagnostic or interventional procedures in the pediatric cardiology cath lab. There are various limitations of this technique e.g. the complexity of application, the absence of standardization and the need of special trained staff for this imaging technique.

MRI and DSCT-scans have better temporal resolution compared to 3D models based on 3DRA. Additionally, MRI- and DSCT-scans can be used for catheter-guidance with new multimodality and overlay registration techniques as well. Thus, these less invasive procedures should be preferred in the daily routine, if available. However, the native rotational angiography includes important flow information with high temporal and spatial resolution. In the hands of an experienced investigator, the immediate availability of these information during the dynamic process of interventional catheterization of complex congenital heart disease, gives 3DRA a great importance in selected cases. The lack of standardization is still an issue.

## Limitations

A limitation is the retrospective nature of our study. The patient collective was a heterogeneous cohort.

The present work was performed in fulfillment of the requirements for obtaining the degree “Dr. med.” at the Friedrich-Alexander-University Erlangen-Nuremberg.
